# Retrospective cohort study investigating extent of pertussis transmission during a boarding school outbreak, England, December 2017 to June 2018

**DOI:** 10.2807/1560-7917.ES.2021.26.26.1900736

**Published:** 2021-07-01

**Authors:** Matt Edmunds, Rachel Mearkle, Jennifer Folliard, Charlotte Anderson, Sooria Balasegaram, Nastassya Chandra, Clare Sawyer, Norman K Fry, Sonia Ribeiro, Gemma Palmer, Michael Morgan, Gill Underhill, Nusreen Ahmad, Simon Friar, Andre Charlett, David Litt, Colin S Brown, Gayatri Amirthalingam

**Affiliations:** 1 UK Field Epidemiology Training Programme, PHE National Infection Service, London, England; 2 Immunisation and Countermeasures Division, PHE National Infection Service, London, England; 3 Thames Valley Health Protection Team, Public Health England, Chilton, England; 4 Field Service, PHE National Infection Service, London, England; 5 Respiratory and Vaccine Preventable Bacteria Reference Unit, PHE National Infection Service, London, England; 6 Boarding school health service, England; 7 PHE Regional Microbiology Laboratory, Southampton General Hospital, Southampton, England; 8 Statistics, Modelling and Economics Department, PHE National Infection Service, London, England; 9 Healthcare Associated Infections and Antimicrobial Resistance Division, PHE National Infection Service, London, England

**Keywords:** Bordetella pertussis, Adolescent, school, intervention

## Abstract

On 1 May 2018, a pertussis outbreak was declared and widespread vaccination recommended at an all-female secondary boarding school in southern England. We conducted a retrospective cohort study to determine the extent of pertussis transmission and identify risk factors in this semi-closed population. Of 504 students and staff assessed before post-exposure vaccination, 48% (n = 240) had evidence of pertussis. A sub-analysis of 409 students found that both residential dormitory (p = 0.05) and school year (p = 0.03) were associated with pertussis, with odds decreasing by 11% for each increase in school year (95% confidence interval: 0.7–20.2). Odds of pertussis were 1.7 times higher in those assumed to have received acellular vaccines for their primary course compared with those assumed to have received whole-cell vaccines (based on date of birth), although this difference was not significant (p = 0.12). Our findings support the need for timely, widespread vaccination following identification of cases among adolescents in a semi-closed United Kingdom (UK) setting and to review the evidence for the introduction of an adolescent pertussis booster to the UK routine vaccination programme.

## Background

Pertussis is a cyclical disease with peaks occurring every 3–4 years [1]. Completion of the accelerated three-dose primary course (at age 2, 3 and 4 months) and preschool booster dose 3 years after the last primary dose (at 3 years and 4 months) in the United Kingdom (UK) was 85.6% in 2017/18 and has been above 80% for the past decade [2]. Despite the high uptake of routine immunisation in England, there was a substantial resurgence of pertussis in 2012, and the incidence of cases observed since then has been considerably higher than in previous years. The most recent epidemic peak occurred in 2016. Similar resurgences have been observed in other countries with longstanding vaccine programmes [3].

After infants under 3 months of age, the next highest incidence of laboratory-confirmed pertussis in the UK is among adolescents aged 10–14 years [1]. There is growing evidence that the shorter duration of protection and lower effectiveness over time against infection conferred by acellular pertussis (aP) vaccines compared with whole-cell pertussis (wP) vaccines have been important contributory factors [4-7]. This is of considerable interest in Europe where a high proportion of countries have switched from a whole-cell to an acellular primary schedule and few routinely recommend adolescent pertussis boosters. In 2018, 35,627 cases were reported in Europe [8]. In the UK, aP vaccines replaced wP vaccines in the accelerated primary course in 2004, later than in many other high-income countries. The single booster dose of aP vaccine has been routinely offered since 2001. Reported increases in adolescent disease, which are in part due to improved case ascertainment but also reflect waning immunity from the childhood programme, has prompted some countries to introduce adolescent boosters. These increases, however, probably underestimate the true burden of disease in adolescent populations [9,10], with delays in recognition and diagnosis being due to atypical presentations. While adolescents are far less likely to experience severe disease compared with infants younger than 3 months, the overall impact on quality of life can be considerable [11] and they are important reservoirs for onward transmission [12].

## Outbreak detection

On 21 March 2018, a local team of Public Health England (PHE) in South England was notified of a serologically confirmed case of pertussis in a 14-year-old student attending an all-female boarding school (school years 7–13, ages 11–18 years). On 1 May, a second confirmed case who resided outside the local team’s jurisdiction was reported, prompting the declaration of an outbreak and initiating a wider investigation. Investigation with the school identified a further two confirmed and one suspected case. The cases were in school years 9 to 13 (ages 14–18 years) and reported onset dates between 25 February and 16 April 2018. As per national guidelines [13], as part of the initial outbreak response, a single dose of pertussis vaccine (Repevax, Sanofi Pasteur, Lyon, France) was considered by the incident management team (IMT) and recommended for students in all affected year groups [9-13] who were residing in boarding houses separate from the younger school years, and to selected staff members. A questionnaire and swabs were taken from individuals attending for vaccination. Clinical evidence of extensive transmission beyond school years 9–13 became immediately apparent and following confirmation by rapid testing, vaccination was extended to all year groups.

This paper summarises the findings of an investigation of a large outbreak of pertussis in a boarding school of 11–18-year-old students. Our objectives were to assess the extent of pertussis transmission within the school setting before post-exposure vaccination by calculating attack rates (AR) of symptomatic and asymptomatic pertussis among students and staff, and to identify risk factors for pertussis.

## Methods

A total of 540 residential and 20 day students attended the school. The residential students lived in 10 dormitories (houses); three accommodating students from years 7 and 8 combined, five with students from years 9 to 11, one for year 12 students and one for year 13 students. In addition to the students, the school had ca 600 staff including teachers, boarding staff, housekeepers and grounds staff.

We conducted a retrospective cohort study of all students and ‘high-risk’ staff. The latter were defined based on their level of exposure and included healthcare staff, boarding staff with regular student contact and staff who were themselves in, or lived with someone in, a priority group according to national guidelines [13].

Active case finding for staff and all year groups was undertaken between 11 and 15 May at the time of the vaccination sessions using a combination of clinical questionnaires, determination of anti-pertussis toxin (anti-PT) IgG titres in oral fluid specimens (OF) and determination of *Bordetella* DNA from throat swabs by PCR. Information on clinical symptoms for each individual was collected using questionnaires at a single point in time. However, active surveillance was implemented at the school for 42 days after symptom onset in the last identified case with a low threshold for testing individuals presenting with cough illness regardless of duration. The local PHE team was also notified of cases linked to the school through routine reporting mechanisms.

### Clinical questionnaire

The questionnaires were administered by boarding school staff. Individuals were asked to provide details on symptoms experienced since January 2018, demographics, accommodation, pre-existing medical conditions and pertussis vaccination history. Based on reported symptoms, individuals were classified into categories of clinical suspicion for pertussis. Definitions for ‘high’ and ‘low’ clinical suspicion were developed by the IMT, based on (but more sensitive than) case definitions in the national pertussis guidelines [13]:

- High clinical suspicion: Cough for 2 weeks or more OR a cough of shorter duration and either vomiting or sore ribs,
- Low clinical suspicion: Onset date within 1 week of completing the questionnaire, and either a cough for less than 2 weeks or two of the following three symptoms: fever, sore throat or tiredness.

For individuals reporting no symptoms, or symptoms not meeting the above criteria, we considered that there was no clinical suspicion for pertussis.

### Microbiological testing

Apart from a small number of serology samples collected before outbreak detection, all samples for microbiological testing were collected by staff at the school health centre. All throat swabs were sent to Southampton Public Health Laboratory for primary PCR testing and all OF and serology specimens were sent to the National Reference Laboratory at PHE Colindale.

### Oral fluid specimens and serology testing

The standard in-house serum anti-PT IgG ELISA reports titres in international units (IU)/mL using a threshold of 70 IU/mL, above which titres are considered indicative of recent infection for samples taken ≥ 2 weeks after onset of cough symptoms [9,14]. The OF assay acts as a surrogate for the serum antibody assay and detects anti-PT IgG but is a capture ELISA and reports in assay arbitrary units (aU), with an equivalent diagnostic threshold of 70 aU [14].

Although the routine diagnostic OF cut-off is > 70 aU for samples taken ≥ 2 weeks after cough onset, in this outbreak, given the uncertainty around sample timing in relation to exposure, the IMT agreed to use a lower threshold of > 60 aU to increase sensitivity. Seronegative samples (≤ 60 aU) known to have been taken 2 weeks or more since symptoms onset were classified as having reliable timing, with all others classified as having unreliable timing.

### PCR testing

PCR detection targeting two *Bordetella pertussis* genomic regions, (i) the pertussis toxin S1 promoter (*ptxP*) and (ii) the insertion element IS*481*, also known to be present in *B*. *holmesii* and some *B*. *bronchiseptica* [15], was conducted on throat swabs (in viral transport medium or dry). We used a TaqMan (Applied Biosystems, Waltham, United States (US)) assay based on Fry et al. [16]. For the purposes of this investigation, a quantification cycle of ≤ 45 for both targets or for the IS*481* target only were both considered as consistent with the detection of *B. pertussis*.

For outbreak management, the following results were considered consistent with *B. pertussis* infection and/or carriage: (i) positive for IS*481* only and (ii) positive for both IS*481* and *ptxP*. Negative samples known to have been taken within 3 weeks of symptom onset were classified as having reliable timing, with all others classified as having unreliable timing.

### Case definitions

Based on test results and clinical suspicion categories, to support the outbreak investigation we created case definitions and calculated AR for asymptomatic and symptomatic individuals. Symptomatic cases were divided into those identified by active case finding vs those identified through routine reporting and further divided into confirmed, probable and possible cases (Table 1).

**Table 1:** Case definitions for symptomatic and asymptomatic cases of pertussis, England, June 2018

Case definition			Description
Symptomatic	Confirmed	Routine reporting	Laboratory-confirmed cases notified to PHE via routine reporting processes
		Active case finding	PCR-positive or anti-PT IgG-positive (serology or OF) AND low or high clinical suspicion
	Probable	Routine reporting	Individuals notified to PHE via routine reporting processes and risk-assessed to be a probable case
		Active case finding	Not PCR-positive or anti-PT IgG-positive (serology or OF) AND absence of a reliable anti-PT IgG-negative result AND high clinical suspicion
	Possible		Not PCR-positive or anti-PT IgG-positive (serology or OF) AND absence of a reliable anti-PT IgG-negative result AND low clinical suspicion
Asymptomatic			Anti-PT IgG-positive (serology or OF) or PCR-positive AND no or unknown clinical suspicion

Anti-PT: anti-pertussis toxin; OF: oral fluid specimen; PHE: Public Health England.

### Vaccination status

Questionnaire responses relating to existing medical conditions and vaccination histories were poorly recorded and not considered suitable for inclusion in the analysis. The extraction of vaccine histories directly from individual medical records was considered but was not logistically feasible, as readily available data were limited and did not include details of type of vaccine given. 

In November 2004, national procurement of vaccines in the childhood programme changed from wP to aP. In the absence of individual vaccination histories, we used date of birth for students born in the UK to infer whether a student would have received aP or wP vaccine for their primary schedule. Allowing an 8-week period from birth to first vaccine, students born on or before 7 September 2004 were inferred to have received wP, with those born later inferred to have received aP. Students born outside the UK were assigned an unknown vaccination status.

For 37 students where school year was not provided, this was calculated based on their date of birth. Date of onset was estimated for 12 confirmed cases based on approximate descriptions (e.g. mid-point of the month used when only month stated).

### Statistical methods

Data were collated and validated in MS Access and analysed using R v1.1.456. Univariate AR (risk ratios) were calculated for each type of case, both overall and by year group. As teachers predominantly lived off-site and as adults would all have been immunised using wP, we used a subset of data including students only. Logistic regression was used to calculate univariate odds ratios for a combined AR incorporating all case definitions, which were calculated for country of birth, year group, house and vaccine type. Sex was not included as all students were female. Multivariable logistic regressions were then conducted, with all variables included a priori because an association was very plausible, to identify the model that best explained the data.

To assess whether participants with high clinical suspicion had statistically different PCR results to those with low or no clinical suspicion, we conducted chi-squared analyses. This was done for individuals where data on symptoms and PCR test with reliable timing were available.

To investigate the impact that the timing of symptom onset in relation to sample collection may have had on differences in allocation of case definitions between younger and older age groups, students with a date of onset and symptoms were dichotomised into Years 7 and 8 and Years 9 to 13 and by whether symptom onset occurred within 21 days of sample collection, and then a two-sided Fisher’s exact test conducted.

### Ethical statement

Informed consent was obtained when taking samples for diagnostic purposes. Public Health England has legal permission, provided by Regulation 3 of The Health Service (Control of Patient Information) Regulations 2002, to process patient confidential information for national surveillance of communicable diseases. As such, individual patient consent was not required for the use of patient data for this outbreak investigation. 

## Results

### Demographics

In total, 504 individuals (409 students and 95 staff) were categorised using the response from the clinical questionnaire and/or microbiological investigations and were included in the analysis. As 560 students were known to attend the school, the student response rate was therefore 73%. A list of all staff considered high-risk was not available, and so it was not possible to calculate a response rate for this group.

All students were female, as were most staff (n = 79; 83%). Most students (n = 295; 72%) and almost all staff (n = 87; 92%) were born in the UK, with over half of non-UK students born in mainland China (n = 23; 20%), Hong Kong (n = 21; 18%) or the US (n = 20; 18%).

### Questionnaire completion

Questionnaires were completed by 453 individuals, 380 of whom were students. Based on reported symptoms, clinical suspicion of pertussis was high for 65 (14%) and low for 125 (28%) of respondents (Table 2). The proportion of respondents with either high or low clinical suspicion varied by school year, with the highest proportion being in Year 12 (36/60) and the lowest in Year 13 (23/65). There was clinical suspicion for 21 of 73 staff completing a questionnaire.

**Table 2:** OF anti-PT IgG test results, PCR test results and clinical suspicion categories for staff and for school years 7 (age 11–12) to 13 (age 17–18), pertussis outbreak, England, December 2017–June 2018 (n = 655)

Data	Outcome	School year (student age in years)														Staff		Total	
		7 (11–12)		8 (12–13)		9 (13–14)		10 (14–15)		11 (15–16)		12 (16–17)		13 (17–18)					
		n	%	n	%	n	%	n	%	n	%	n	%	n	%	n	%	n	%
Clinical suspicion	High	4	11	4	16	8	14	10	14	7	11	12	20	8	12	12	16	65	14
	Low	10	28	7	28	23	40	22	30	15	24	24	40	15	23	9	12	125	28
	None	22	61	14	56	27	47	42	57	40	65	24	40	42	65	52	71	263	58
	Total	36	100	25	100	58	100	74	100	62	100	60	100	65	100	73	100	453	100
	Unknown	3		39		31		32		23		26		26		22^a^		202	
OF anti-PT IgG test result	Positive	12	32	3	12	15	25	18	24	25	40	10	16	11	15	6	6	100	21
	Reliable negative	0	0	2	8	10	17	10	14	7	11	14	22	14	20	11	12	68	14
	Unreliable negative	26	68	20	80	34	58	46	62	30	48	40	63	46	65	76	82	318	65
	Total	38	100	25	100	59	100	74	100	62	100	64	100	71	100	93	100	486	100
	No test	1		39		30		32		23		22		20		2^a^		169	
PCR test result	Positive	7	18	3	12	8	13	13	17	3	5	4	6	8	11	19	20	65	13
	Reliable negative	2	5	6	24	12	19	19	25	2	3	17	27	6	8	14	15	78	16
	Unreliable negative	29	76	16	64	43	68	45	58	61	92	43	67	58	81	60	65	355	71
	Total	38	100	25	100	63	100	77	100	66	100	64	100	72	100	93	100	498	100
	No test	1		39		26		29		19		22		19		2^a^		157	

Anti-PT: anti-pertussis toxin; OF: oral fluid specimen.

^a^ Total number of high-risk staff not known, figures represent identified staff with missing data.

### Laboratory testing

Of 486 individuals tested for anti-PT IgG in OF, 100 (21%) had evidence of recent infection (> 60 aU titre), 68 (14%) a negative result with reliable timing, and 318 (65%) a negative result with unreliable timing. Among students assessed by OF test (70%; 393/560), the highest proportion with recent infection was in Year 11 (25/62) and the lowest in Year 8 (3/25). There was no clear trend between year groups. Of 93 staff assessed by OF test, six (6%) had evidence of recent infection.

Of 498 individuals who had a throat swab for pertussis PCR testing, 65 (13%) were PCR-positive, 78 (16%) had a negative result with reliable timing and 355 (71%) a negative result with unreliable timing. Three of the positive results were both IS*481*- and *ptxP*-positive, the remaining 62 were positive for IS*481* only. Among students providing a throat swab (72%; 405/560), the highest proportion of PCR-positive students was found in Year 7 (7/38) and the lowest in Year 11 (3/66). There was no clear trend between year groups. Of 93 staff assessed by PCR test, 20 (20%) were PCR-positive.

### Association between PCR test result and symptoms

Data on symptoms and a PCR test with reliable timing was available for 137 participants. The proportion of positive test results was higher among participants who had high clinical suspicion of pertussis compared with those for whom there was low (chi-squared p = 0.02) and no (chi-squared p < 0.01) clinical suspicion (Table 3).

**Table 3:** Cross-tabulation of clinical suspicion category against PCR test result, pertussis outbreak in a school, England, December 2017–June 2018 (n =137)

Clinical suspicion	PCR test result				Total
	Positive		Negative (reliable)		
	n	%	n	%	n
High	12	55	10	45	22
Low	17	27	45	73	62
Sub-clinical / none	30	57	23	43	53
Total	59	43	78	57	137

### Attack rates

Of the 504 individuals assessed, a total of 72 were confirmed (AR: 14%), 19 probable (AR: 4%) and 59 possible (AR: 12%) symptomatic cases. Of the confirmed and probable cases, only eight of 72 and two of 19, respectively, were identified by routine reporting.

In addition, there were 90 asymptomatic individuals (AR: 18%). Questionnaires were unavailable for 15 individuals classified as asymptomatic. These could potentially have been categorised as symptomatic cases, had questionnaires been available.

The combined AR (symptomatic and asymptomatic) was 48% (n = 240) (Table 4). For students, the highest combined AR was for Year 7, with the lowest in Year 13. The overall AR in staff was lower than all student year groups (n = 33; 35%). Overall AR for UK-born (n = 183; 48%) and non-UK-born individuals (n = 57; 47%) were similar. The median ages were similar (Wilcoxon rank sum, p = 0.05) for combined student cases (15 years) and student non-cases (16 years).

**Table 4:** Pertussis attack rates by case type for school years 7 (ages 11–12) to 13 (ages 17–18) and staff, school outbreak, England, December 2017–June 2018 (n = 504)

Group		Case definition										Non-case		All in study	Students with no data
		Symptomatic						Asymptomatic		Combined					
		Confirmed		Probable		Possible									
		n	%	n	%	n	%	n	%	n	%	n	%		
School year	7	8	21	0	0	6	16	9	24	23	61	15	39	38	1
	8	4	16	2	8	4	16	2	8	12	48	13	52	25	39
	9	13	19	3	4	12	18	7	10	35	52	32	48	67	22
	10	14	18	2	3	11	14	16	21	43	56	34	44	77	29
	11	11	17	0	0	6	9	21	32	38	58	28	42	66	19
	12	8	13	5	8	10	16	5	8	28	44	36	56	64	22
	13	6	8	4	6	5	7	13	18	28	39	44	61	72	19
All students		64	16	16	4	54	13	73	18	207	51	202	49	409	151
Staff		8	8	3	3	5	5	17	18	33	35	62	65	95	^a^
**Total**		**72**	**14**	**19**	**4**	**59**	**12**	**90**	**18**	**240**	**48**	**264**	**52**	**504**	**^a^**

^a^ Total number of high-risk staff not known.

### Outbreak progression

Date of onset was available for 90 of 150 (60%) cases meeting possible, probable and confirmed case definitions (Figure). Of 90 asymptomatic cases, 50 tested IgG-positive and PCR-negative, indicating that exposure may have been more than 3 weeks before sampling. Based on the PCR sampling date, 43 individuals may have been exposed earlier than 20 April 2018 (Figure). Two cases were identified after vaccination through active case finding.

**Figure:**
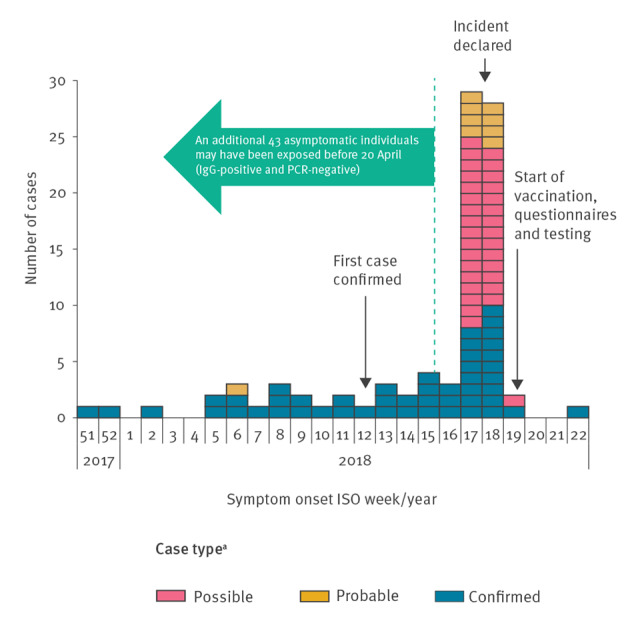
Pertussis cases by case definition and week of disease onset, school outbreak, England, December 2017–June 2018 (n = 90)

ISO: International Organization for Standardization.

^a^ See Table 1 for details.

There were 144 students with a date of onset and symptoms (including 35 that did not meet the definition for high or low clinical suspicion), 13 in Years 7–8 and 131 in Years 9–13. The two-sided Fisher’s Exact test indicated (p = 0.02) that the proportion of students tested more than 21 days after symptom onset was lower in Years 7–8 (n = 2; 15%) than in older groups (n = 65; 50%).

### Risk factors for pertussis

Univariate analysis of school year as a linear variable showed a significant relationship with pertussis (p = 0.03), with the odds of pertussis in students decreasing by 11% for each increase in school year (95% confidence interval: 0.7–20.2). House as a categorical variable was also significantly associated (chi-squared statistic, p = 0.05), with odds ranging from 0.58 to 2.33. Odds of pertussis were 1.7 times higher in those receiving aP vaccines as the primary course compared with those receiving wP vaccines, although the association between vaccine type and pertussis was not significant (chi-squared statistic, p = 0.12). Birth region showed no association with pertussis (chi-squared statistic, p = 0.66) and was excluded from further models. Univariate logistic regression model outputs are shown in Table 5.

**Table 5:** Univariable logistic regression models for students, pertussis school outbreak, England, December 2017–June 2018 (n = 409)

Variable	Group	Cases	Non-cases	Odds	OR	p	95% CI
School year	School year	NA			0.89	0.03	0.80–0.99
House (p = 0.05^a^)	House A	13	14	0.93	Reference		
	House B	13	10	1.30	1.40	0.56	0.46–4.36
	House C	7	3	2.33	2.51	0.24	0.57–13.64
	House D	30	13	2.31	2.49	0.07	0.92–6.87
	House E	17	14	1.21	1.31	0.61	0.46–3.72
	House F	23	20	1.15	1.24	0.66	0.47–3.28
	House G	14	24	0.58	0.63	0.36	0.23–1.71
	House H	24	15	1.60	1.72	0.28	0.64–4.72
	House I	29	37	0.78	0.84	0.71	0.34–2.09
	House J	25	40	0.62	0.67	0.39	0.27–1.67
	Unknown	12	12	1.00	1.08	0.89	0.36–3.26
Birth region (p = 0.66)	United Kingdom	154	141	1.09	Reference		
	Africa	5	2	2.50	2.29	0.33	0.48–16.16
	Asia	30	36	0.83	0.76	0.32	0.44–1.30
	Europe	5	8	0.62	0.57	0.34	0.17–1.76
	North America	10	12	0.83	0.76	0.54	0.31–1.82
	Other	3	3	1.00	0.92	0.91	0.17–5.02
Vaccine type (p = 0.30)	Whole cell	124	123	1.01	Reference		
	Acellular	30	18	1.67	1.65	0.12	0.88–3.17
	Unknown	53	61	0.87	0.86	0.51	0.55–1.34

CI: confidence interval; NA: not applicable; OR: odds ratio;

^a^ p value significant but rounded up.

Bivariable and multivariable models were built using all combinations of school year, house and vaccine type. There was considerable collinearity between these variables. Years 7 and 8 lived in houses A to C, Years 9 to 11 lived in houses D to H, Year 12 lived in house I and almost all of Year 13 (65/68) lived in house J. Based on date of birth, all students in school Years 7 and 8 were assumed to have received a primary course using aP vaccine, with students in older school years assumed to have received a primary course of wP vaccine. This precluded the use of multivariable models in determining which group of variables best predicted whether a child had pertussis. The univariable models were therefore considered the most reliable for inference for risk factors of pertussis.

## Discussion

This study evaluates the extent of symptomatic and asymptomatic pertussis transmission in an outbreak setting using questionnaires, PCR and OF anti-PT IgG testing. A strength of this investigation is the collection of demographic and laboratory data from individuals who reported no symptoms. This is different than during most outbreak investigations, when only symptomatic individuals are swabbed. Our findings demonstrate a rate of confirmed symptomatic disease (AR: 14%) similar to those previously reported in other pertussis school outbreaks [17,18]. When including all symptomatic (AR: 30%) and asymptomatic cases (AR: 18%), the rate more than tripled (AR: 48%). Given the low proportion of cases identified through routine reporting, this study provides valuable insight into the potential magnitude of pertussis under-ascertainment.

Our findings of higher AR in younger age groups might be unexpected given that immunity wanes following childhood vaccination and the initial reported cases were in the older students. However, in the UK, cohorts born before 2004 were eligible to receive wP vaccines only as part of the primary infant schedule and after 2004, only aP containing vaccines were recommended. Thus, younger cohorts were fully primed and boosted with aP vaccines only. As there is evidence that disease onset tended to be later in Years 7 and 8, it is unlikely that the higher symptomatic AR in younger groups was due to insufficient time for clinical symptoms to develop in older age groups at the time questionnaires were administered.

PCR testing targeting a repetitive sequence in *B. pertussis,* conducted as part of another boarding school outbreak in Sydney, Australia [19], found no association with symptomatic cases. However, a positive PCR result appeared more likely when specimens were collected close to the date of symptom onset, including some (n = 6) that were positive before onset [19]. In our study, a larger proportion of individuals with a PCR result from a sample taken within 3 weeks of symptom onset tested positive in those with high clinical suspicion, compared with those with low or no clinical suspicion, providing further evidence of this.

Our finding that AR were 1.7 times higher in students assumed to have received aP vaccine than in those assumed to have received wP vaccine based on their date of birth suggests that aP vaccine offers a shorter duration of protection, although our study was not sufficiently powered to demonstrate statistical significance (p = 0.12). Differences in respiratory hygiene and mixing patterns may also have played a role.

Owing to the high degree of collinearity between house and school year, it is not possible to determine which was the most important exposure, although both were significant (p = 0.05 and p = 0.03, respectively. The p value of 0.05 was rounded up, and so this was significant). There is good plausibility for either of these variables to impact odds of pertussis through increased risk of transmission in classroom and/or residential settings.

Consideration of post-exposure vaccination is recommended in the national public health guidelines in England [13]. While there is limited published evidence of effectiveness, the rationale for such an approach is to rapidly boost antibody levels in a susceptible population to reduce ongoing transmission. There is good scientific plausibility for this, and a similar approach has been adopted in the US [20].

While this was not a randomised intervention, which would have enabled a more robust evaluation of the use of vaccination in an outbreak setting, the prompt reduction in cases (only two identified after vaccination) suggests that vaccination is likely to have helped control the outbreak. The generalisability of this study to adolescent populations is restricted by the fact that the study population was almost exclusively female, where behaviour may be different from mixed populations. However, the elevated levels of pertussis transmission demonstrated through this unique investigation highlight the importance of timely post-exposure vaccination where cases are identified in semi-closed settings, and demonstrate the susceptibility in these age groups.

While routine immunisation against pertussis has helped reduce pertussis morbidity in childhood, there is evidence it may not have a notable impact on the incidence of adolescent and adult pertussis, with incidence in these age groups increasing in some countries [21]. The World Health Organization recommends that countries consider the need for additional booster doses taking into account local epidemiology [7] but the age at which boosters are offered requires careful consideration, as an adolescent booster will shift the peak incidence of pertussis towards groups of child-bearing age [22]. Our findings suggest there may be an argument for the introduction of an adolescent pertussis booster in the UK programme.

Our study had some limitations. Information on clinical symptoms and samples for testing for each individual were collected at a single point in time as it was not logistically feasible to follow up progression of symptoms (except for the two cases detected by active surveillance). This is likely to have affected allocation of case definitions in the following ways: (i) Any individuals with disease onset within 2 weeks of questionnaire completion may have been categorised as possible instead of probable because they did not have time to meet the 2-week cough criteria; (ii) asymptomatic carriers who were exposed within 2 weeks of being tested may have tested positive for OF anti-PT IgG had the test been conducted after 2 weeks from exposure; this would have resulted in them being counted as asymptomatic cases; (iii) there was a reduced likelihood of a true case testing positive and hence being categorised as confirmed or asymptomatic.

Because of the age of our study population and the lack of individual vaccine histories (including product information), it was not possible to identify whether the aP vaccine effectiveness waned over time, as pupils from only two school years had received the aP pertussis vaccine. The lower AR observed in Year 8 is likely to be due to 20 students from this year group spending January to April overseas on a field trip, and so having a lower level of exposure. Although they only made up a small proportion of the total cohort, it was not possible to determine from the available information whether data were provided for day students and if so, which students these were. The lower exposure risk for these students means they have the potential to confound our results if associated with any of our exposure variables. While the response rate for students was high (73%), as no information was available for students not included in the study, it is not known what effect selection bias may have had on our findings. Another limitation of this study is the inability to discern the direction of transmission, in particular whether asymptomatic individuals transmitted infection to others.

## Conclusion

Our findings support the need for timely, widespread vaccination following identification of cases among adolescents in a semi-closed (UK) setting, to review the evidence for the introduction of an adolescent pertussis booster to the UK routine vaccination programme and, in the longer term, advocate for better vaccines that have a longer period of protection.
